# Clinical and prognostic features of cerebral toxoplasmosis in HIV infected patients – a case series

**DOI:** 10.1186/1471-2334-13-S1-P10

**Published:** 2013-12-16

**Authors:** Andrei Vâță, Carmen Manciuc, Cristina Nicolau, Liviu Prisacariu, Carmen Dorobăț

**Affiliations:** 1 “Gr.T.Popa” University of Medicine and Pharmacy, Iaşi, Romania; 2 Regional HIV/AIDS Center Iaşi, Romania

## Background

Although cerebral toxoplasmosis incidence has declined as a result of antiretroviral treatment (ART), it still is a serious diagnostic and therapeutic problem and a life-threatening condition.

## Methods

We performed a retrospective study of the medical charts of patients diagnosed with cerebral toxoplasmosis admitted to the Infectious Diseases Hospital of Iaşi in the last 5 years (2008-2012).

## Results

Cerebral toxoplasmosis led to the diagnosis of HIV infection in 3/11 patients, and was the AIDS defining disease in 2/11 cases. Most of the patients (9/11) belonged to the historical 1989 cohort; the mean age was 22.5 years, 6/11 were males. The mean CD4+ cell count was 26.7 cells/cmm (95%CI: 5.5-49.7); all the patients were viremic at the time of diagnosis (mean viral load: 1.21x10^5^ copies/mL). The clinical manifestations varied, ranging from mild persistent headache to coma. Neurologic manifestations were seen in 7/11 of cases, 5/11 had convulsions. All patients were seropositive for IgG antibody against *Toxoplasma gondii* in blood, but only 5/11 had high titers (>300 UI/mL). Cerebral imaging studies (6 CT and 5 IRM) were performed and showed suggestive lesions in all cases. 7/11 patients were receiving ART before diagnosis, and 6/11 had recommendations for co-trimoxazole prophylaxis, but most of them had a low adherence the treatment. Co-trimoxazole was the main anti-toxoplasma drug in all cases; clindamycin or clarithromycin were sometimes associated (2/11). 4/11 patients died, three of them being newly discovered, late presenter HIV cases. 2 patients had relapses and 4/11 neurological sequelae.

## Conclusion

Cerebral toxoplasmosis is a rare but serious opportunistic infection, seen more frequently in severely immunocompromised, non-adherent patients, but it can be sometimes seen in previously apparently healthy individuals.

